# Mechanism of internal browning of pineapple: The role of gibberellins catabolism gene (*AcGA2ox*) and GAs

**DOI:** 10.1038/srep33344

**Published:** 2016-12-16

**Authors:** Qin Zhang, Xiuwen Rao, Lubin Zhang, Congcong He, Fang Yang, Shijiang Zhu

**Affiliations:** 1 Guangdong Province Key Laboratory of Postharvest Physiology and Technology of Fruits and Vegetables, College of Horticulture, South China Agricultural University, Guangzhou 510642, Guangdong Province, China; 2 South Subtropical Crops Research Institute, Chinese Academy of Tropical Agricultural Sciences/Hainan Key Laboratory for Postharvest Physiology and Technology of Tropical Horticultural Products, Zhanjiang 524091, Guangdong Province, China

## Abstract

Internal browning (IB), a physiological disorder (PD) that causes severe losses in harvested pineapple, can be induced by exogenous gibberellins (GAs). Over the years, studies have focused on roles of Gibberellin 2-oxidase (GA2oxs), the major GAs catabolic enzyme in plants, in the regulation of changes in morphology or biomass. However, whether GA2oxs could regulate PD has not been reported. Here, a full-length *AcGA2ox* cDNA was isolated from pineapple, with the putative protein sharing 23.59% to 72.92% identity with GA2oxs from five other plants. Pineapples stored at 5 °C stayed intact, while those stored at 20 °C showed severe IB. Storage at 5 °C enhanced *AcGA2ox* expression and decreased levels of a GAs (GA_4_) ‘compared with storage at 20 °C. However, at 20 °C, exogenous application of abscisic acid (ABA) significantly suppressed IB. ABA simultaneously upregulated *AcGA2ox* and reduced GA_4_. Ectopic expression of *AcGA2ox* in *Arabidopsis* resulted in reduced GA_4_, lower seed germination, and shorter hypocotyls and roots, all of which were restored by exogenous GA_4/7_. Moreover, in pineapple, GA_4/7_ upregulated polyphenol oxidase, while storage at 5 °C and ABA downregulated it. These results strongly suggest the involvement of AcGA2ox in regulation of GAs levels and a role of AcGA2ox in regulating IB.

Internal browning (IB), also referred to as blackheart, is a physiological disorder (PD) of pineapple (*Ananas comosus* [L.] Merr.) commonly occurring during storage and shipment, which results in severe loss of commercial value^1^. Exogenous gibberellins (GAs) treatment induces IB^1^,^2^,^3^, while low temperature storage inhibits it^2^,^4^,^5^. Low temperature (4 °C) increases the level of bioactive GAs in *Arabidopsis* seeds^6^ but reduces GAs levels and inhibits growth of *Arabidopsis* seedlings^7^. How low temperature influences GAs biosynthesis in harvested pineapple remains unclear.

GAs are endogenous phytohormones that are involved in the regulation of the life cycle of plants, including seed germination, leaf expansion, stem elongation, floral induction and fruit maturation^8^. The major bioactive GAs, such as GA_1_ and GA_4_, are synthesized from trans-geranylgeranyl diphosphate by the sequential actions of cyclases, membrane-associated mono-oxygenases and soluble 2-oxoglutarate-dependent dioxygenases^9^. The dioxygenases catalyze the later steps of GA biosynthesis, including the removal of the C-20 group and the introduction of the 3β-hydroxyl group. Negative feedback is a central mechanism of GAs biosynthesis, such as the GA 20-oxidase genes and a GA 3β-hydroxylase gene of *Arabidopsis*, both of whose transcript levels are subject to feedback regulation^10^, indicating their roles in the maintenance of the endogenous levels of bioactive GAs^11^.

The GAs catabolism is another important factor that regulates the endogenous levels of bioactive GAs^11^. In many plant species, bioactive GAs and their immediate precursors are 2β-hydroxylated by GA 2-oxidase (GA2ox) to produce biologically inactive products^12^. Genes encoding GA2ox s were first identified in 1999^13^,^14^,^15^. Over the years, the functions of GA2oxs have been well-documented. Overexpression of *GA2ox* genes caused decreases in levels of bioactive GAs and dwarfing phenotypes in rice^11^, poplar^16^, *Arabidopsis*^17^ and wheat^18^. In fact, by limiting bioactive GA content, they regulate development at various stages during the plant life cycle. They prevent seed germination in the absence of light and cold stimuli, delay the vegetative and floral phase transitions, limit the number of flowers produced per inflorescence, suppress elongation of the pistil prior to fertilization^19^, influence underground biomass growth of *Populus*^20^, and affect fruit set and growth in tomato^21^. Our recent work suggested that downregulation of a GA2ox could be related to the delay of senescence of a harvested leafy vegetable^22^. However, to the authors’ knowledge, there has not been any study on the role GA2oxs in regulating PD. IB is one of the most typical and devastating PDs of pineapple whose occurrence is related to application of GAs^1^,^2^,^3^. Therefore, IB is an ideal PD for addressing the role of GA2oxs. Studying *AcGA2ox* gene in the context of IB would surely add to the understanding of the functions of *GA2oxs* gene family.

Here through displaying the expression patterns of *AcGA2ox* gene in response to a series of treatments that regulate GA levels and IB development in pineapple, in combination with the phenotypes connected with deficiency in bioactive GAs of *Arabidopsis* lines overexpressing *AcGA2ox*, we show that enhanced expression of *AcGA2ox* resulted in lower bioactive GA levels and thus suppressed IB development.

## Results

### Cloning and Characterization of *AcGA2ox* cDNA

**Figure 1:**
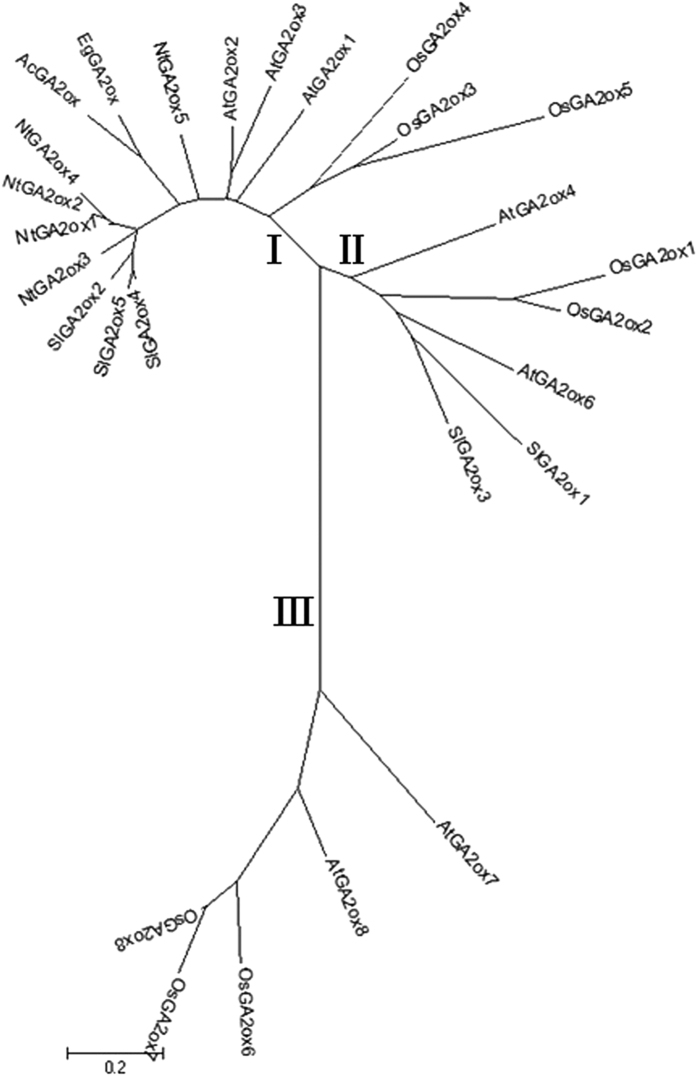
Maximum-likelihood phylogenetic tree based on comparison between pineapple GA2ox (AcGA2ox) protein sequence and GA2ox proteins from five other species, i.e., Arabidopsis (*Arabidopsis thaliana*), rice (Oryza sativa), tomato (*Solanum lycopersicum*), tobacco (*Nicotiana tabacum*) and oil palm (*Elaeis guineensis*). The phylogenetic tree was created using MEGA6.06 (downloaded from the website http://www.megasoftware.net/mega.php). The three GA2ox classes (I, II, and III) are indicated. Protein names and the corresponding Genbank accession numbers of the proteins are: AcGA2ox(ACN30002), AtGA2ox1(CAB41007), AtGA2ox2(CAB41008), AtGA2ox3(CAB41009), AtGA2ox4(AAG51528), AtGA2ox6(AAG00891), AtGA2ox7(AAG00891), AtGA2ox8(ABE66080), OsGA2ox1(BAB40934), OsGA2ox2(BAC16751), OsGA2ox3 (BAC16752), OsGA2ox4 (AAU03107), OsGA2ox5(BAC10398), OsGA2ox6 (CAE03751), OsGA2ox7(BAG98459), OsGA2ox8(XP_006648857), SlGA2ox1(EF441351), SlGA2ox2(EF441352), SlGA2ox3(EF441353), SlGA2ox4(EF441354), SlGA2ox5(EF441355), NtGA2ox1(BAD17855), NtGA2ox2(BAD17856), NtGA2ox3(ABO70985), NtGA2ox4(AGL39429), NtGA2ox5(ABO70986), and EgGA2ox(AFS65097.1).

By means of homologous cloning and RACE-PCR, the full-length ***AcGA2ox*** cDNA of 1367 bp was isolated, with an open reading frame consisting of 981 bp, encoding a protein of 326 amino acids and designated *AcGA2ox* (GenBank accession number FJ645911) (Fig. S1). Sequence analysis shows that the putative AcGA2ox protein, with molecular weight of 36.2 kD, and a pI of 5.95 according to Compute pI/MW program, contains amino acids that are conserved for GA2oxs, including His-113, Asp-115, and His-272 (Fig. S1) in the 2OG-FeII_Oxy domain. These residues are thought to associate at the catalytic site and bind with Fe^2+^ 23. In addition, the POSORT and MotifScan analyses show that AcGA2ox has six protein kinase C phosphorylation sites and six casein kinase II phosphorylation sites, similar to GA2oxs from other plants. Alignment of the putative pineapple AcGA2ox with all the known GA2oxs from *Arabidopsis*, rice, tomato, tobacco and oil palm showed identities between 23.59% and 72.92%%. AcGA2ox shares the highest identity (72.92%) with the GA2ox from monocot oil palm (Table S1) and the phylogenetic analysis showed that AcGA2ox falls into GA2ox protein class I and is clustered in the same sub-group with EgGA2ox (Fig. 1), while showing identities between 25.79% and 58.88% with the four GA2oxs from rice, also a monocot. On the other hand, AcGA2ox shares very high identity with all the five GA2oxs from the dicot tobacco, ranging from 58.12% to 60.06%. However, AcGA2ox shows the lowest identity with GA2ox protein class III AtGA2ox7 and AtGA2ox8, 23.59% and 26.35%, respectively (Table S1). Transient expression of AcGA2ox in protoplasts of *Arabidopsis* mesophyll indicates that it was localized in cytoplasm (Fig. S2).

### AcGA2ox expression is related to IB development in pineapple

**Figure 2:**
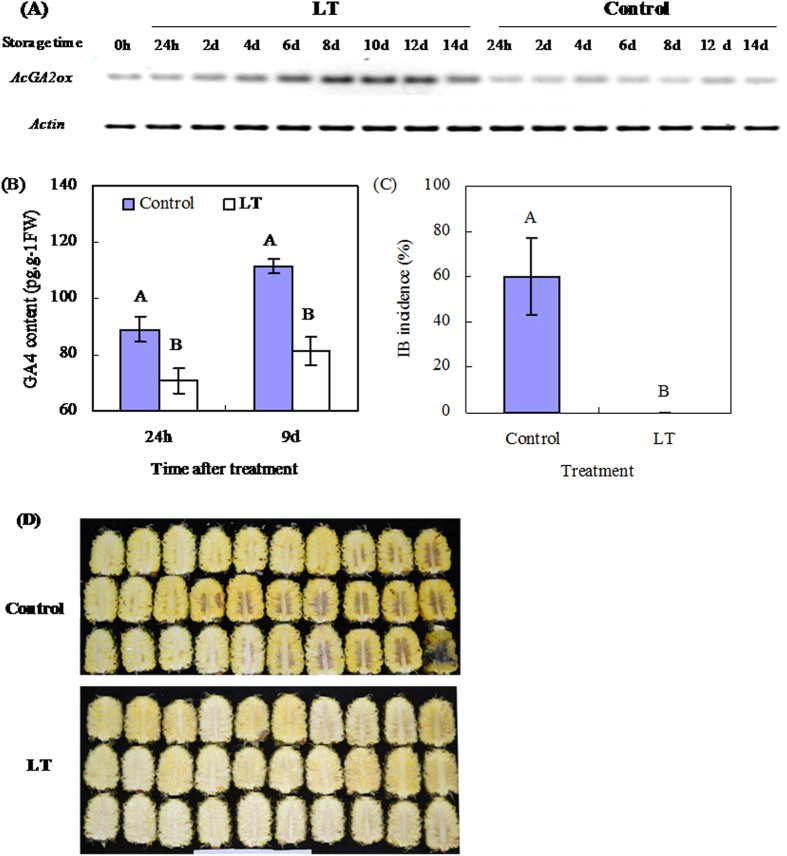
Effects of low temperature on *AcGA2ox* gene expression, endogenous GA_4_ levels, and IB development. For LT treatment, pineapple fruits were stored at 5 °C, with those stored at 20 °C serving as control. *AcGA2ox* gene expression in pulp of pineapple fruits was analyzed by semi-quantitative RT-PCR (**A**) while GA_4_ levels in the pulp were determined by ELISA (**B**). IB incidences (**C**) were evaluated and the pictures of symptoms (**D**) taken after 14 d of storage. The significance of observed differences between the control and LT treatment is indicated by a letter above the relevant bar (P ≤ 0.01). Standard errors are shown (**B**,**C**).

**Figure 3:**
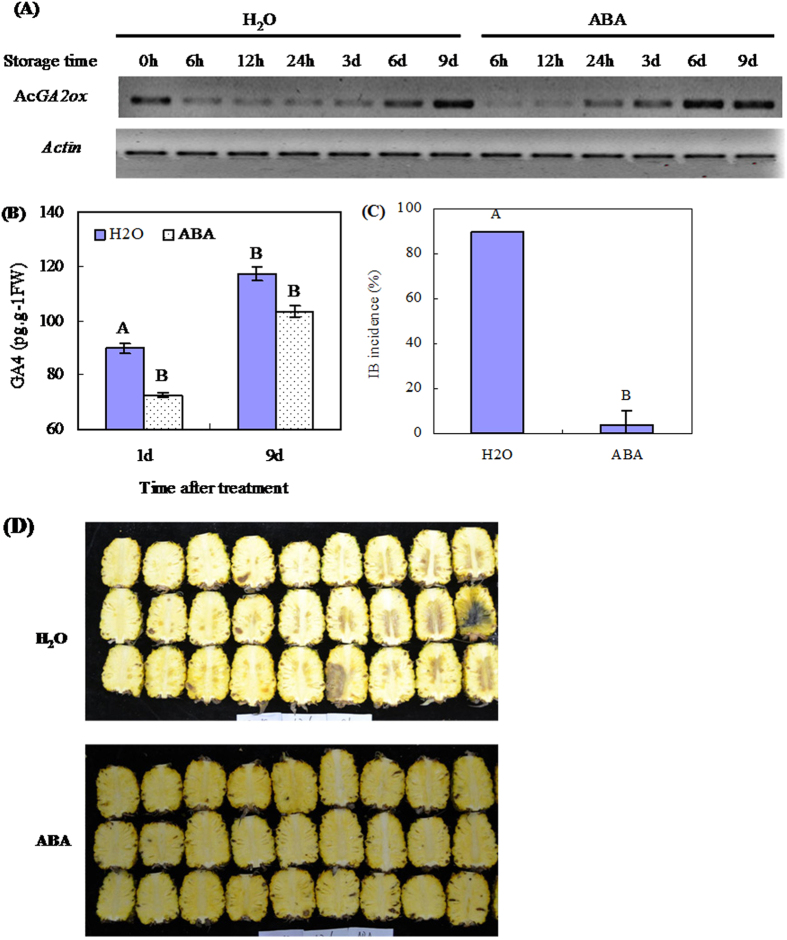
Effect of ABA on *AcGA2ox* gene expression, endogenous GA_4_ levels, and IB incidence in harvested pineapple fruits. Fruits were sprayed with ABA solution at 200 mg.L^−1^, with distilled water as control. Following treatment, both ABA-treated and control fruit were stored at 20 °C. *AcGA2ox* gene expression in pulp of pineapple fruits was analyzed using semi-quantitative RT-PCR (**A**) while GA_4_ levels in the pulp were determined by ELISA (**B**). IB incidences (**C**) were evaluated and the pictures of symptoms (**D**) taken after 14 d of storage. Significant differences between the control and LT treatment are indicated by letters above each bar (P ≤ 0.01). Standard errors are shown (**B**,**C**).

Firstly, low temperature (LT, 5 °C) upregulates *AcGA2ox* and inhibits IB development in pineapple. LT enhances *AcGA2ox* transcript accumulation (Fig. 2A), and reduces GA_4_ levels by 26% on 1 day after treatment (DAT) and 37% on 9 DAT compared with the control (20 °C) (Fig. 2B), as measured by RT-PCR. Meanwhile, pineapples stored at LT for 14 d remain intact, while the control shows severe symptoms of IB (Fig. 2C,D). Secondly, exogenous abscisic acid (ABA) application enhances *AcGA2ox* transcription at ambient temperature (20 °C) (Fig. 3A), and decreases endogenous GA_4_ by 24% on 1 DAT and 14% on 9 DAT (Fig. 3B). At the same time, ABA treatment significantly inhibits IB incidence in pineapple fruits (Fig. 3C,D). However, exogenous GA_4/7_ treatment upregulates *AcGA2ox*, but the GAs biosynthesis inhibitor paclobutrazol downregulates it (Fig. S3).

### *AcGA2ox* over-expressing *Arabidopsis* display phenotypes related to GAs deficiency

**Figure 4:**
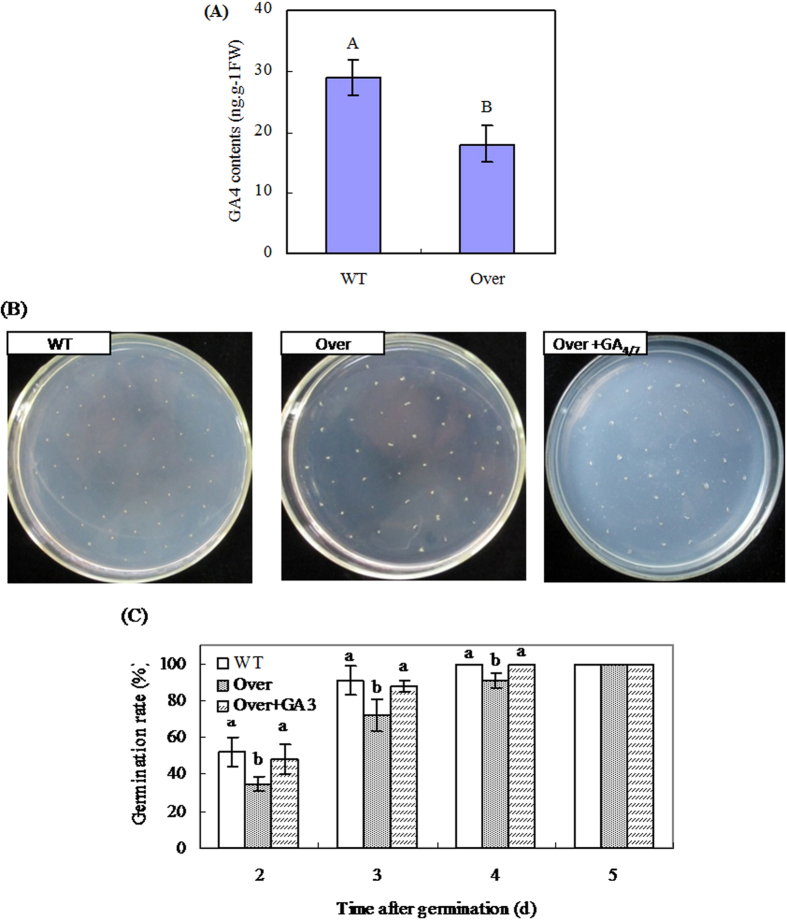
GA_4_ contents in *AcGA2ox* over-expression *Arabidopsis* (**A**) and effects of exogenous GA_4/7_ on germination of seeds transgenic lines (**B**,**C**). GA_4_ levels in the seedlings were determined by ELISA 7 d after germination. For assay of seed germination, sterilized T3 transgenic seeds were sown on the MS culture medium, either complemented with GA_4/7_ (Sigma) at 25 mg.L^−1^ or without. Significance of differences is indicated by letters above the bars (P ≤ 0.05). WT: wild type of Arabidopsis. Over: transgenic *Arabidopsis* overexpresing *AcGA2ox* gene. Standard errors are shown (**A**,**C**).

**Figure 5:**
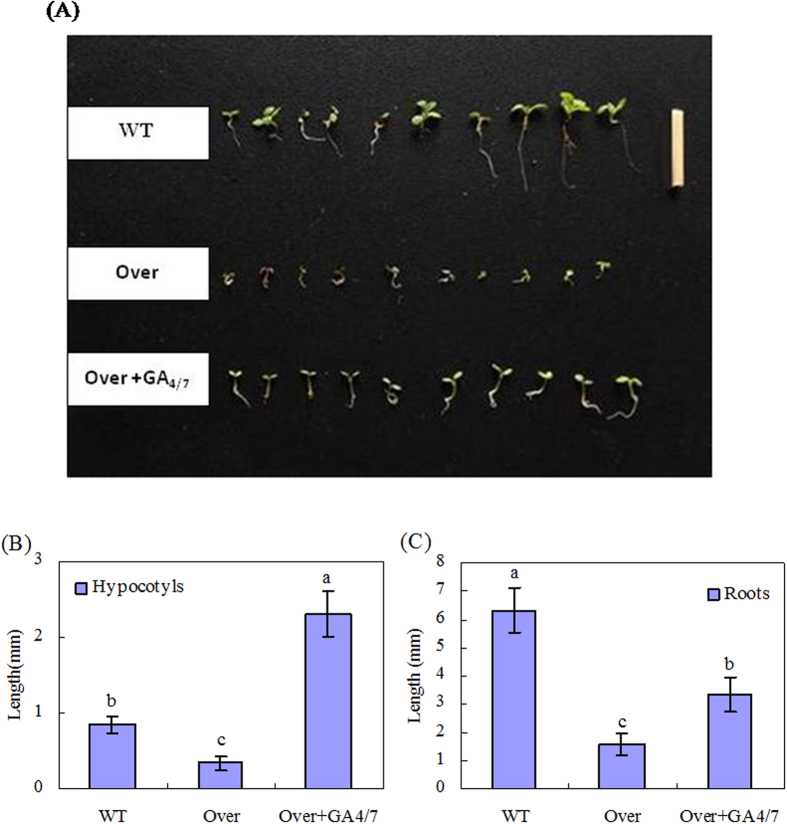
Length of hypocotyls and roots of transgenic *Arabidopsis* seedlings over-expressing *AcGA2ox* in response to GA_4/7_. GA_4/7_ at 25 mg.L^−1^ were applied by adding to the MS culture medium. Picture (**A**) was taken and length of hypocotyls and roots length (**B**,**C**) measured 15 d after germination. WT: wild type of Arabidopsis. Over: transgenic Arabidopsis overexpresing *AcGA2ox* gene. The scale bar on the upper right is 10 mm. Significant differences are indicated by letters above the bars (P ≤ 0.05). Standard errors are shown (**B**,**C**).

For ectopical expression in *Arabidopsis,* the 978 bp cDNA of *AcGA2ox* was cloned into the pCAMBIA1305.1 vector downstream of a CaMV 35S promoter (Fig. S4), which was then used to transform *Arabidopsis* plants, generating transgenic *Arabidopsis* lines stably overexpressing *AcGA2ox* (Fig. S5). The transgenic plants over-expressing *AcGA2ox* have significantly lower GA_4_ content (Fig. 4A) and seed germination rates (Fig. 4B,C) than wild-type *Arabidopsis* plants. However, when cultured on GA_4/7_-containing medium, the transgenic seeds show significantly higher germination rates (*P* ≤ 0.05), comparable to that of the wild type (Fig. 4B,C). In addition, the hypocotyl and root length of the transgenic line is significantly decreased compared to wild type, which is partly restored by exogenous GA_4/7_ (Fig.5A–C). In addition, *AcGA2ox* is constitutively expressed in transgenic plants, but not in wild type (Fig. S6), and *AtGA3ox1* and *AtGA5* are up-regulated in the transgenic plants (Fig. S6). These results confirm the function of *AcGA2ox* in regulating GAs levels.

### Polyphenol oxidases expression in response to factors affecting *AcGA2ox* gene expression and IB

**Figure 6:**
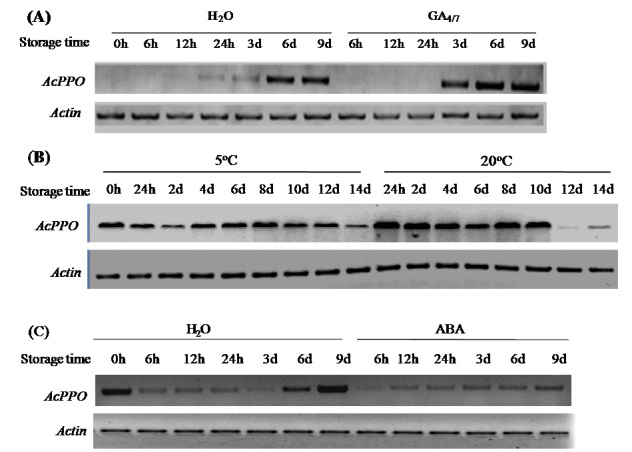
Semi-quantitative PCR showing gene expression of polyphenol oxidase (PPO) in pulp of pineapple fruits in response to treatment with GA_4/7_, low temperature, and ABA. For LT treatment, pineapple fruits were stored at 5 °C, with those stored at 20 °C serving as control. Fruit surfaces were treated with either GA_4/7_, ABA, or distilled water. Following treatment, all fruits were stored at 20 °C.

As activities of polyphenol oxidases (PPO) were correlated with the degree of browning^2^, expression of polyphenol oxidase (*PPO*) in pineapple fruits was investigated. The results show that GA_4/7_ treatment enhances *AcPPO* expression, while low temperature and ABA decreases *AcPPO* expression (Fig. 6). This confirms that the *AcGA2ox* gene plays an important role in regulating IB in pineapple through the modulation of GAs levels.

## Discussion

GA2oxs are encoded by a multigene family^15^. Here we cloned a GA2ox from pineapple, an herbaceous and monocot plant. The protein sequence of AcGA2ox includes His-113, Asp-115, and His-272 in the 2OG-FeII_Oxy domain (Fig. S1). These residues are thought to associate with Fe^2+^ 23 at the catalytic site and are conserved in all known rice GA2ox proteins^24^. Although the sequence identity between AcGA2ox and 26 GA2oxs from five different plant species varies greatly, AcGA2ox shares the highest identity with (72.92%, Table S1), and clustered in the same sub-group as, EgGA2ox from oil palm (Fig. 1).

The *GA2ox* family is composed of two subfamilies differing in their substrate specificity, C_19_-GAs and C_20_-GAs^17^. C_19_-GA 2-oxidation is a major GA inactivation pathway in *Arabidopsis*^19^. C_19_ -GA2oxs hydroxylate the C-2 of active C_19_-GAs (GA_1_ and GA_4_) or C19-GA precursors (GA_20_ and GA_9_) to form biologically inactive GA_8_, GA_34_, GA_51_, and GA_29_, respectively^24^,^25^, while C_20_ -GA2oxs, a more recently discovered type of GA2oxs which includes AtGA2ox7 and AtGA2ox8, use C_20_-GAs as substrates^17^. Class I & II belong to the C_19_ -GA2oxs subfamily, while class III belongs to the C_20_ -GA2oxs subfamily^21^. Phylogenetic analysis shows that AcGA2ox falls into class I (Fig. 1), suggesting that it inactivates GAs by hydroxylating C_19_-GAs such as GA_1_ and GA_4_. AcGA2ox is localized in cytoplasm (Fig. S2) and both C_20_-GAs and C_19_-GAs are formed in cytoplasm^26^, which enables AcGA2ox to act quickly in response to the changes in internal and external environments.

Studies show that the expression of GA2ox in response to LT could differ strikingly. LT (4 °C) suppresses expression of *AtGA2ox2* and increases the level of bioactive GAs in *Arabidopsis* seeds^6^, but LT upregulates expression of *GA2ox* genes in *Arabidopsis* seedlings, reducing GA levels and inhibiting the seedling growth^7^. Exposure to LT increases expression of *PsGA2ox2* in pea plants^27^. Most importantly, under those cases, GA2oxs all function by negatively regulating the concentration of bioactive GAs. In this study, we show that LT (5 °C) upregulates expression of *AcGA2ox* and decreases levels of GA_4_, one of the major bioactive forms of GAs (Fig. 2), consistent with former studies on the mechanism of GA2oxs in regulating GAs.

Previous studies showed that GA_3_ application leads to development of IB in pineapple^1^,^3^. In this study, we further showed that ABA, the antagonist of GAs, effectively inhibits IB development in pineapple fruits stored in ambient temperature. Furthermore, exogenous ABA enhances *AcGA2ox* expression and reduces GA levels (Fig. 3). These results are significant for three reasons. Firstly, they confirm that bioactive GAs exceeding a certain level cause IB. Therefore, to keep physiologically healthy, a plant or an organ of plant should tightly maintain the concentration of bioactive GAs. Perhaps this is why exogenous GAs reduces the expression of *GA20ox* genes and stimulates the expression of *GA2ox* genes in rice^11^ and enhances the expression of *GA2ox* genes in *Arabidopsis*^15^,^19^. These reports are consistent with our results that GA_4/7_ quickly activates expression of *AcGA2ox* in pineapple fruits, which is just as quickly inactivated by paclobutrazol, an inhibitor of GAs biosynthesis^28^,^29^ (Fig. S3). This mechanism could also explain why overexpressing *AcGA2ox* from pineapple in *Arabidopsis* plants causes the *Arabidopsis* genes *AtGA5* and *AtGA3ox1* to be upregulated (Fig. S6): in order to tightly maintain GAs levels. Secondly, since GAs concentrations are regulated at both the level of hormone synthesis and through controlled inactivation, GA 2-oxidation is a major inactivating pathway for the plant hormone GAs^19^. These results also suggest that, in harvested pineapple, ABA antagonizes GAs partly by inactivating biosynthesis of active GAs. Thirdly, these results confirmed that, in harvested pineapple, AcGA2ox plays an important role in negatively regulating active GAs.

The roles of GA2oxs in different processes of plant development have been extensively elucidated. These processes include fruit growth^21^, underground growth^19^, seed germination, and vegetative and reproductive transitions^20^. Dwarfing is the most typical phenotype of plants overexpressing *GA2oxs*, such as *AtGA2ox7* and *AtGA2ox8* in *Arabidopsis* and tobacco^17^, poplar *PtaGA2ox1*^16^ and *PtGA2ox5*^20^ in poplar trees, spinach *SoGA2ox3* in *Nicotiana sylvestris*^30^, plum *PslGA2ox* in *Arabidopsis*^31^, and grapevine *VvGA2ox* genes in *Arabidopsis*^32^. However, apart from morphological changes, there has been no report showing what happens to the plants or plant organs that no longer grow when GA2ox expression is upregulated. Here we showed that the enhanced *AcGA2ox* expression in harvested pineapple in response to low temperature and ABA is related to the inhibition of IB (Figs 2 and 3). Since gene transcript levels do not necessarily correspond to functional activity of enzymes, *Arabidopsis* overexpressing *AcGA2ox* was generated in order to show how the pineapple *AcGA2ox* gene regulates GAs levels (Figs S4 and S5). We showed that the transgenic plants overexpressing *AcGA2ox* display decreased GA_4_ levels, lower seed germination rates (Fig. 4), and shorter hypocotyls and roots (Fig. 5) than wild-type plants. All of these phenotypes are reversed by application of GA_4/7_. This strongly suggests that the phenotypes are a result of GAs deficiency. Our results are consistent with the phenotypes of *Nicotiana sylvestris* overexpressing *GA2ox3* from spinach^30^ and the response of *Arabidopsis overexpressing AcGA2ox* to GAs is similar to that of rice overexpressing GA2ox6^25^. These results confirm that *AcGA2ox* negatively regulates bioactive GAs concentration in harvested pineapple.

IB involves oxidation of phenolic compounds, and activities of polyphenol oxidases (PPO) were correlated with the degree of browning^2^,^33^. Promoters of two pineapple polyphenol oxidase (*PPO*) genes (*PINPPO1* and *PINPPO2*), when transiently expressed in pineapple fruits and ectopically expressed in tobacco, were GA_3_ responsive^1^. Here we showed that the *AcPPO* gene was upregulated in response to GA_4/7_ (Fig. 6A) and downregulated in response to LT and ABA (Fig. 6B,C), suggesting that *AcGA2ox* regulates IB development by influencing active GAs levels in harvested pineapple. This is the first report showing the link between *AcGA2ox* and a physiological disorder.

In conclusion pineapple AcGA2ox reduces levels of bioactive GAs, leading to decreased transcription of *AcPPO*, which then results in decreased oxidation of phenolic compounds and contributes to inhibition of IB, the major physiological disorder of pineapple.

## Methods

### Plant materials and treatment

Pineapple (*Ananas comosus* [*L.*] Merr. cv ‘Comte de Paris’) fruits at commercial maturity were collected from a commercial plantation in Xuwen County, Guangdong Province and stored at low temperature (5 °C), with fruits stored at 20 °C as control. Sample pulp tissues were collected at 6 h, 12 h, 24 h and every 2 d or 3 d thereafter, frozen in liquid nitrogen and stored at −80 °C.

For ABA or GA_4/7_ treatment, solutions of ABA at 200 mg.L^−1^ or GA_4/7_ at 300 mg.L^−1^ was sprayed on the surface of pineapple until runoff. The control fruits were sprayed in the same way with distilled water. Following treatment, pineapples were stored at 20 °C. Samples of pulp tissues were collected at 6 h, 12 h, 24 h, 3 d, 6 d, and 9 d, frozen in liquid nitrogen and stored at −80 °C.

### RNA preparation

Total RNA from pulp of pineapple was extracted following the method of Wan and Wilkins (1994)^34^ and RNA from *Arabidopsis thaliana* plants was extracted using Trizol reagent (Invitrogen, USA) according to the supplier’s instruction. Contaminant genomic DNA was digested by RNase-free DNaseІ.

### Isolation of *AcGA2ox* full-length cDNA

First strand cDNA was synthesized using RevertAid^TM^ First Strand cDNA Synthesis Kit according to the manufacturer’s protocol. cDNA was used as a template for amplifying a partial sequence of the *AcGA2ox gene*. The forward and reverse primers were 5′-GGA(G) TTC TTC(T) AAA(G) GTC (GT) A(G)TA(CGT) AAC(T) CA -3′ and 5′-TA(CT)A CA(G)C TCT T A(G)A AC(T)C TC(T)C CA(G)T T-3′, respectively. Conditions for PCR amplification: 35 cycles of 94 °C for 1 min, 45 °C for 1.5 min and 72 °C for 2 min.

The 5′- and 3′- ends of the *AcGA2ox* cDNA were obtained by RACE-PCR, using the 5′-Full RACE Kit and the 3′- RACE version2.0 (TaKaRa, Shiga, Japan). For the 5′-RACE, the primers for the 1st nested PCR and the 2nd nested PCR amplifications were: 5′-AGA GCT CCT TCT CGA CCT GTG GCA-3′ (2 × 2-5outer), 5′-ACC TCA ATG CTT CGT CCT CCA AC-3′ (2 × 2-5inner), respectively. Those for 3′-RACE were 5′-TCT GTT CCT CCT GAT CAA AGC TCT-3′ (2 × 2-3outer), 5′-TCT TCA TCA ATG TTG GCG ATT CA-3′ (2 × 2-3inner), respectively. The PCR products were separated in 1.0% (w/v) agarose gels, purified and ligated into the pMD-18T vector (TaKaRa, Shiga, Japan). The nucleotide sequences of the cDNA inserts were analyzed using the thermo sequenase dye terminator cycle sequencing kit and a 377 DNA sequencer (Perkin-Elmer Applied Biosystems).

### Sequence analysis

DNA sequence data were analyzed using the programs provided by the National Centre of Biotechnology (NCBI) web site (http://www.ncbi.nlm.nih.gov). The BLASTN and BLASTP programs were used for the gene sequence homology search. Amino acid sequences of selected plant GA-2oxidase genes were aligned using ClustalW2 (http://www.ebi.ac.uk/Tools/msa/clustalw2/). A phylogenetic tree was constructed using maximum parsimony with 500 bootstrap replicates of MEGA6.06. ExPASy web site (http://cn.expasy.org/tools/) and programs therein were used for the prediction of amino acid features of AcGA2ox.

### Gene expression analysis

Semi-quantitative RT-PCR was used for the analysis of gene transcript accumulation in pineapple fruits (*AcGA2ox* and *AcPPO*) and leaf tissues of *Arabidopsis* transgenic lines (*AcGA2ox, AtGA5, AtGA3ox1*) following the method of Matsushita *et al.*^35^ and Zhu *et al.*^36^. The primers (Table 1) were designed according to cDNA sequences. The *actins* from pineapple (*AcActin*) and *Arabidopsis* (*AtActin*) were PCR amplified as an internal control (Table 1).

### Observation of IB severity

Three fruits from each treatment, one from each replicate, were cut every three days for monitoring the development of IB. Upon obvious onset of browning in flesh or core of pineapples from any treatment, all the remaining fruits (10–12 fruits per replicate) were then cut for observation of IB. IB incidence was calculated as percentage of fruits with symptoms of IB.

### Determination of GA_4_ contents in pineapple tissue and Arabidopsis

GA_4_ from pineapple and *Arabidopsis* was extracted and purified according the method described by Talon and Zeevaart^37^. The total concentrations of GA_4_ were then determined following the method by Yang *et al.*^38^ using ELISA kits manufactured by Jiang Lai Bio-Technology, Shanghai, China)^39^.

### Generation of overexpression construct of ***Ac**GA2ox*

For ectopic expression of the *AcGA2ox* gene, the coding sequence was transferred to pCAMBIA1305.1 (kindly provided by prof. Yaoguang Liu from Key Laboratory of Plant Functional Genomics and Biotechnology, Education Department of Guangdong Province, South China Agricultural University, Wushan Road, Guangzhou 510642, China) at the *Spe I* and *Pst I* sites using T4 ligase (Invitrogen, USA), fused with 35S promoter. The construct pC-*AcGA2ox* was then transferred into *Agrobacterium tumefaciens* strains LBA4404 and transformed into *Arabidopsis* using the floral dip method^40^.

### Screening of transgenic lines

*Arabidopsis* seeds from plants subjected to floral dip transformation were surface sterilized, sown onto 1% agar containing MS medium containing kanamysin (50 μg.ml^−1^) and selected for transgenic seedlings according the protocol described by Clough and Bent^40^. One week after germination, transformants had green and expanded cotyledons, and extended roots, while non-transformants showed yellow cotyledons and under-developed roots and died soon. The kanamycin-resistant transformants identified were then transplanted and grown in growth chambers under long days (16 h light/8 h darkness) and the day and night temperatures were 22 °C and 16 °C, respectively, for production of T1 seeds.

### Transgene detection

DNA and mRNA extracted from the kanamycin-resistant transformants and T1, T2 and T3 plants were used as templates to perform PCR using *AcGA2ox* gene-specific primers *AcGA2ox-*F (5’-GACTAGTTCAAAATTGCCCAAGCCT-3’) and *AcGA2ox-*R (5’-AACTGCAGACCTCAACAACAACCATG -3’). All PCR products were visualised on a 1.1% agarose gel containing ethidium bromide (1 μg.ml^−1^).

### Phenotype analysis of transgenic Arabidopsis plants over-expressing pineapple *AcGA2ox* gene

Seed germination was assayed following the method by Laval *et al.*^41^ with adaption. Sterilized T3 transgenic seeds were sown on MS culture medium, either complemented with GA_4/7_ (Sigma) at 25 mg.L^−1^or without. Seeds were incubated 2 d at 4 °C prior to the transfer to culture chamber at 22 °C with 16/8 h light/dark photoperiod. Germination was scored within 4 d of incubation. Length of hypocotyls was measured 15 d after germination.

### Experiment design and statistical analysis

The experiments were completely randomized designs. Treatments were applied to three replications of 20 fruit for IB evaluation, three replications of 100 seeds for seed germination assay, and three replications of 20 seedlings for measurement of hypocotyls and root length. Data were analyzed by one-way analysis of variance (ANOVA). Mean separations were performed using the least significant difference method (LSD test). Statistically significant differences were assumed when their *P* values were ≤0.05 and very significant differences when P ≤ 0.01. Different letters above the bars in the figures indicate significance at the 0.05 level (with lower case letters) or 0.01 level (with upper case letters).

## Additional Information

**How to cite this article**: Zhang, Q. *et al.* Mechanism of internal browning of pineapple: The role of gibberellins catabolism gene (*AcGA2ox*) and GAs. *Sci. Rep.* **6**, 33344; doi: 10.1038/srep33344 (2016).

## Supplementary Material

Supplementary Information

## Figures and Tables

**Table 1:** Sequences of Primers for Semi-quantitative RT-PCR.

Gene	Specific Primers used for Semi-quantitative RT-PCR	
	Forward primer (5′–3′)	Reverse primer (5′–3′)
*AcGA2ox*	CAAAATTGCCCAAGCCT	ACCTCAACAACCACCATG
*AtGA5*	CTCGTGTATTCATGAGCGTCTGA	GCCTGTAAGAAGCTTTCT
*AtGA3ox1*	CCATTCACCTCCCACACTCT	GCCAGTGATGGTGAAACCTT
*AcPPO*	AGTGCCTGGTTTAGGTGTATT	TGATGGTGGATTGGTATGG
*AcActin*	ATGGCAGACGGAGAGGATATTCA	GCCTTTGCAATCCACATCTGCTG
*AtActin*	ATCCGGAAAGTACCCAGAT	AAGAACCATGCACTCATCAGC
