# Supplementary material for: Mechanism of internal browning of pineapple: The role of gibberellins catabolism gene (AcGA2ox) and GAs
Source: Sci Rep. 2016 Dec 16;6:33344. doi: 10.1038/srep33344 (PMC5159799; doi:10.1038/srep33344)
Supplement: Supplementary Information [file srep33344-s1.pdf]

Mechanism of internal browning of pineapple: The role of gibberellins catabolism gene (*AcGA2ox*) and GAs

Qin Zhang, Xiuwen Rao, Lubin Zhang, Congcong He, Fang Yang & Shijiang Zhu

Supplementary data Figure S1

```
1     gaaattcaccaccaatatcaagccccttcctctgttttcccctgtttcccccctgttt
61    ccttctttacctcaacaacaaccATGGTGGTGCTGGCGCACGGAGAGCTAGAGCAAATC
1                             M  V  V  L  A  H  G  E  L  E  Q  I
121   TCTCTCCCAGTGGTGCACAAACCCACCAACTTCTTCTCCGATGTCCCGGTCATCGACCTC
13     S  L  P  V  V  H  K  P  T  N  F  F  S  D  V  P  V  I  D  L
181   TCCGATCCCGAGTCGAAAGCGCGGCTCATCGGCGCGTGCGAGGAGCTCGGGTTCTTCAAG
33     S  D  P  E  S  K  A  R  L  I  G  A  C  E  E  L  G  F  F  K
241   GTGACCAACCATGGGATCCCAATGGAGCTCATGGCTAAGTTGGAGGACGAAGCATTGAGG
53     V  T  N  H  G  I  P  M  E  L  M  A  K  L  E  D  E  A  L  R
301   TTCTTCTCATTGCCACAGGTCGAGAAGGAGCTCTCTGCTTCTTCCCCAGGGCCTTTCGGC
73     F  F  S  L  P  Q  V  E  K  E  L  S  A  S  S  P  G  P  F  G
361   TACGGGAGTAAGAAAATCGGGGGAAATGGTGATCTGGGCTGGGTGGAGTACATCCTTTTG
93     Y  G  S  K  K  I  G  G  N  G  D  L  G  W  V  E  Y  I  L  L
421   GAGATCACATCAAAGCCTATTTCTCATTCCTCATTATCCTTTCTTAAGGAACCCTCAGCA
103    E  I  T  S  K  P  I  S  H  S  S  L  S  F  L  K  E  P  S  A
481   AGCTTCTCGGTCTTGAATGAGTACATCACTGCTGTGAGAAAACTAGCTTGTGAGGTGTTA
123    S  F  S  V  L  N  E  Y  I  T  A  V  R  K  L  A  C  E  V  L
541   GAATTAATGGCGGAGAGCCTGAAAATTCAACCAAAGAATGTGTTCAGCAAATTGGTCACG
143    E  L  M  A  E  S  L  K  I  Q  P  K  N  V  F  S  K  L  V  T
601   GATTCGGAGAGCGACTCGATGTTGCGGCTCAACCACTACCCGCCATGCCCACACCTTCAA
163    D  S  E  S  D  S  M  L  R  L  N  H  Y  P  P  C  P  H  L  Q
661   GGCCTGGATTGTGGCTTGACTGGGTTTGGAGAACACACTGACCCCCAGATTATATCTTTG
183    G  L  D  C  G  L  T  G  F  G  E  H  T  D  P  Q  I  I  S  L
721   CTGAGGTCCAACAACACCTCAGGATTGGAGATTTCACTGAGGGATGGGAGTTGGGTCTCT
203    L  R  S  N  N  T  S  G  L  E  I  S  L  R  D  G  S  W  V  S
781   GTTCCTCCTGATCAAAGCTCTTTCTTCATCAATGTTGGCGATTCATTGCAGGTTCTGACA
223    V  P  P  D  Q  S  S  F  F  I  N  V  G  D  S  L  Q  V  L  T
841   AATGGAAGATTCAGGAGCGTAAAACACCGGGTTCTGGCAAACAGTGCGCAATCGCGGGTG
243    N  G  R  F  R  S  V  K  H  R  V  L  A  N  S  A  Q  S  R  V
901   TCGATGATATACTTCGGGGGACCGTCGCCGAGAGAGAGGCTGGCGCCATTGCCGCTGTTG
263    S  M  I  Y  F  G  G  P  S  P  R  E  R  L  A  P  L  P  L  L
961   ATAGAAGAAGGGAAGCAGAGCCTCTACAGGGAGTTCACATGGTGCGAGTACAAGAGATCT
283    I  E  E  G  K  Q  S  L  Y  R  E  F  T  W  C  E  Y  K  R  S
1021  GCATACAAAACAAGGTTGTCTGATAATAGGCTTGGGCAATTTTGAaatgtgagggaaaaa
303    A  Y  K  T  R  L  S  D  N  R  L  G  Q  F  *
1081  gttcatgtagttaaatttcagaaaaaagaaagcaaagtaagtaggttttgaaaagaggag
1141  gtctccaaagtttagttgctaagtgcagttgccaaagatggaaacatagaggttctttta
1201  atttactctctctctctctctctttttattggtttttttgcttcctattttttgcccccctct
1261  ctttctctcttcctttcttgtgtctaattacactggttcctttcatgcaatggaagtttc
1321  ttttgaatataacatgaatagcttagtttgcttctgaaaaaaaaaa
```

Figure S1. Nucleotide and deduced amino acid sequences of *AcGA2ox*. Start codon is in bold and italics, stop codon is indicated by an asterisk. Gene-untranslated regions are shown in lower case, 2OG-FeII_Oxy domain is shaded by gray background, and conserved amino acid is shown in a rectangular box. Numbers on the left margins correspondingly represent nucleotide and deduced amino acid sequences.

Supplementary data Figure S2

(A)

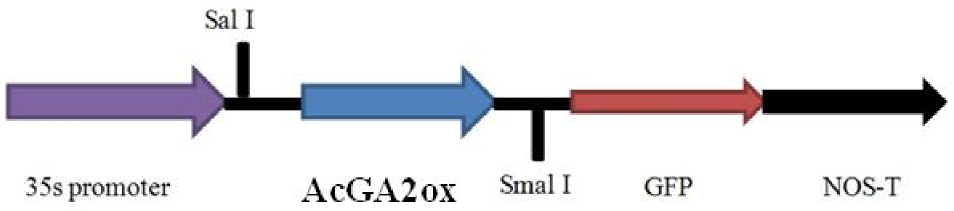

(B)

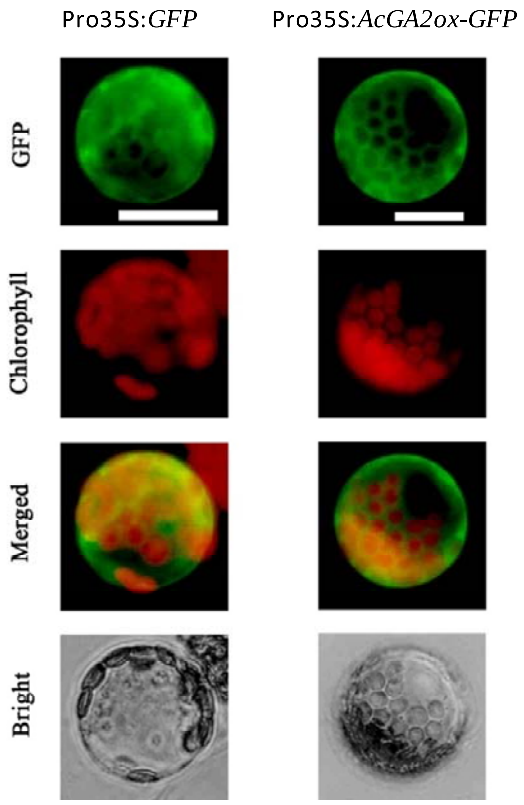

Figure S2. Transient expression of AcGA2ox in *Arabidopsis* mesophyll protoplast. (A) Expression vector construction; (B) Fluorescence microscopy showing the sub-cellular localization of AcGA2ox protein. The coding sequence of *AcGA2ox* was cloned as a C-terminal fusion in-frame with green fluorescent protein (GFP) into pEZS-NL vector, driven by the CaMV 35S promoter. Protoplasts obtained from *Arabidopsis* mesophyll were transfected. —50μm size bar.

Supplementary data Figure S3

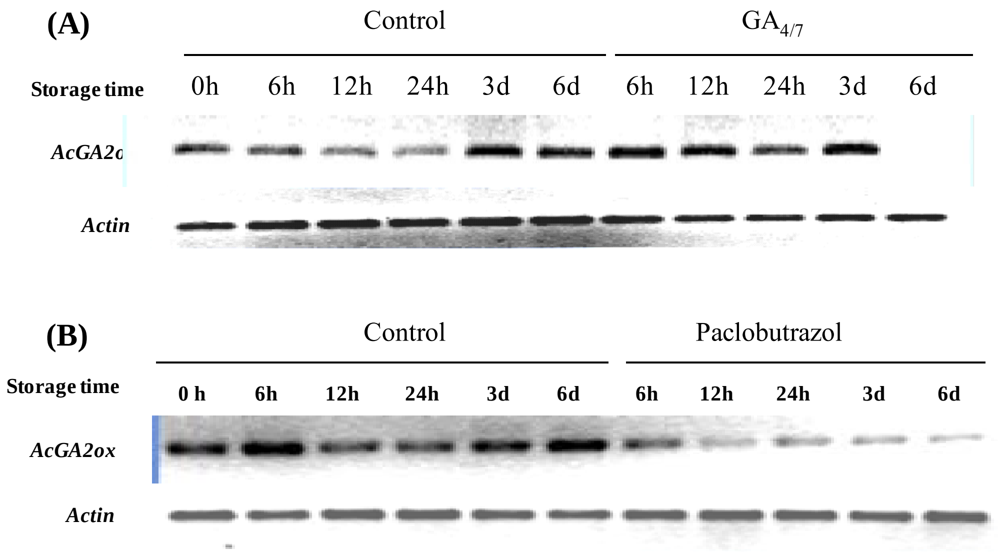

Figure S3 Effect of exogenous application of $GA_{4/7}$ and paclobutrazol on expression of *AcGA2ox* gene in pulp of pineapple. Solutions of GA_3 at 300 $mg.L^{-1}$ or paclobutrazol at 200 $mg.L^{-1}$ was sprayed to harvested pineapple fruits. The control was sprayed in the same way with distilled water. Following treatment, fruits were stored at 20 ^{o}C.

Supplementary data Figure S4

(A) (B)

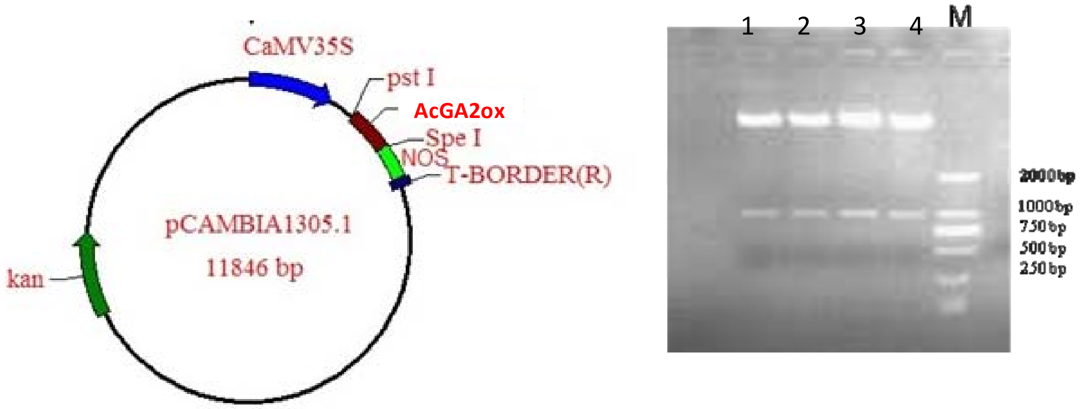

Figure S4. pC-AcGA2ox overexpression vector construction and verification. (A), The 978 bp full-length *AcGA2ox* sequence was cloned into pCAMBIA1305.1 downstream of CaMV 35S promoter to construct pC-AcGA2ox, which was used to transform *E.coli*. Kan was used to screen for positive clones. (B), Digestion of the extracted plasmid DNA from the positive clones with Spe I and Pst I gave a specific band of 978 bp and sequencing showed no mutation in basic and right orientation. Lane 1-4, digestion of the recombinant DNA by enzymes; Lane M, marker.

Supplementary data Figure S5

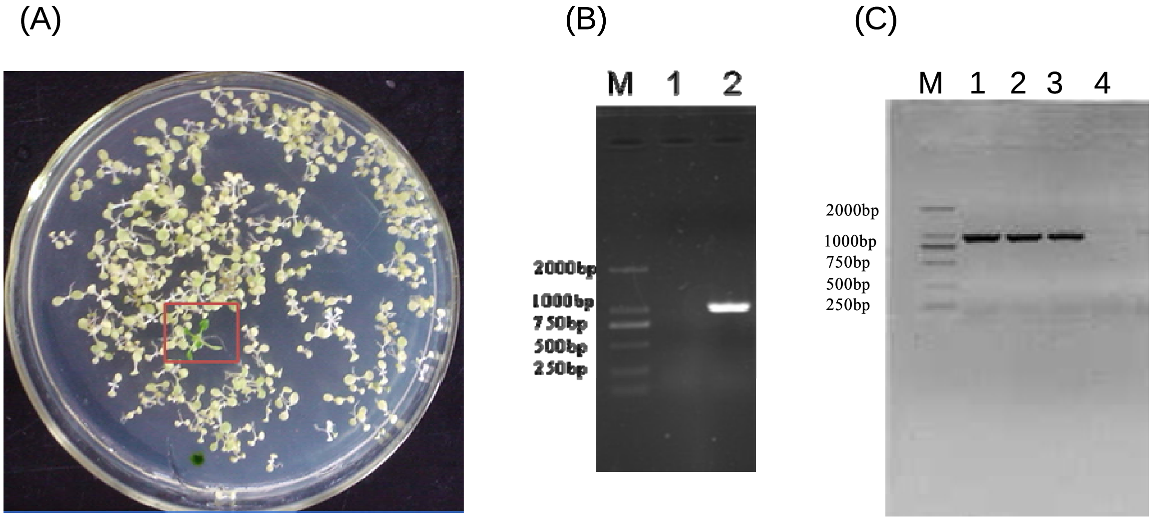

Figure S5. Screening for *Arabidopsis* plants over-expressing *AcGA2ox*. (A), Seeds from T0 generation were harvested and sown on MS medium containing 50 mg.ml^{-1} kanamycin，cultured in a growth chamber under artificial light for 14 d. (B), PCR using the sequences at both ends of *AcGA2ox* as specific primers against genomic DNA from the plantlet with green leaves and extended roots (marked by red-line box) in (A) and from wild type plants showing that *AcGA2ox* was integrated into T1 (B) and T3 (C) generation *Arabidopsis* plant. M, marker; lane 1 in (B) and line 4 in (C), wild type plant; lane 2 and line 1-3 in (C), T1 and T3 generation, respectively.

Supplementary data Figure S6

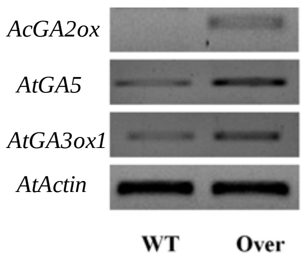

Figure S6. Expression intensity of two *Arabidopsis* genes (*AtGA5* and *AtGA3ox1*) involved in GA biosynthesis in leaves of *Arabidopsis* overexpressing *AcGA2ox* gene from pineapple. Gene expression of *Arabidopsis* seedlings was analyzed 7 d after germination. Gene expression was analyzed with semi-quantitative RT-PCR. WT: wild type of *Arabidopsis*. Over: transgenic *Arabidopsis* overexpresing *AcGA2ox* gene.

Supplementary data Table S1 Comparison of pineapple GA2ox (AcGA2ox) with GA2oxs from five other plant species. Values indicate percentage identity, which were obtained by using ClustalW2 (http://www.ebi.ac.uk/Tools/msa/clustalw2/). See Fig. 1 for accession nos.

	1	2	3	4	5	6	7	8	9	10	11	12	13	14	15	16	17	18	19	20	21	22	23	24	25	26	27
1: AcGA2ox																											
2: EgGA2ox	72.92																										
3: OsGA2ox5	58.88	55.38																									
4: OsGA2ox3	57.55	56.11	56.51																								
5: OsGA2ox4	54.75	55.61	54.34	68.35																							
6: AtGA2ox2	52.35	54.66	46.42	51.75	52.34																						
7: AtGA2ox3	50.00	47.96	44.51	48.08	50.00	69.25																					
8: NtGA2ox2	58.65	56.51	45.89	52.12	49.31	55.80	56.19																				
9: SlGA2ox2	58.58	57.69	45.69	52.96	51.40	54.72	55.91	89.62																			
10: NtGA2ox1	60.00	59.13	46.91	53.48	53.10	56.71	55.25	90.09	87.15																		
11: NtGA2ox4	58.12	55.11	45.68	52.53	51.33	55.49	55.25	85.14	80.88	86.75																	
12: SlGA2ox4	59.06	58.82	47.22	53.48	52.23	56.27	55.42	81.62	85.17	84.85	76.36																
13: SlGA2ox5	59.19	57.41	47.69	53.00	51.54	56.50	54.74	82.97	84.06	83.73	77.71	89.12															
14: NtGA2ox3	59.44	57.36	45.85	50.78	50.66	54.10	54.77	80.06	80.50	80.61	76.36	78.48	76.74														
15: NtGA2ox5	60.06	60.74	48.62	54.23	53.16	59.82	55.59	64.29	63.95	68.58	65.86	63.83	64.76	62.35													
16: AtGA2ox1	58.15	56.01	46.86	51.78	53.39	55.86	51.70	56.23	56.91	58.39	55.90	57.50	55.73	57.01	55.35												
17: OsGA2ox1	40.25	41.56	39.81	41.21	44.02	37.43	38.72	39.75	40.32	41.72	39.88	40.31	39.33	41.72	38.39	39.44											
18: OsGA2ox2	41.64	41.69	44.58	43.59	44.49	39.04	39.14	40.82	40.45	41.85	41.54	39.81	39.76	40.31	41.19	38.89	63.09										
19: AtGA2ox6	44.26	43.65	40.58	45.67	46.30	44.27	45.16	47.52	46.84	48.08	46.47	47.28	45.86	45.19	46.62	45.45	49.54	46.63									
20: SlGA2ox3	46.93	47.91	40.57	47.37	46.51	43.40	43.95	49.19	51.14	50.63	47.48	49.53	47.96	47.95	47.17	46.35	42.40	44.74	55.25								
21: SlGA2ox1	48.01	47.37	41.80	45.12	42.86	44.62	44.27	49.34	49.50	51.13	48.87	50.16	47.44	49.68	47.65	44.94	41.18	42.39	54.14	53.66							
22: AtGA2ox4	47.59	47.95	42.47	45.96	44.81	45.97	48.48	51.56	51.04	51.34	50.34	50.17	50.17	51.01	48.83	47.49	47.95	45.81	52.46	48.73	50.47						
23: OsGA2ox7	25.79	23.32	25.68	24.60	26.45	24.80	24.00	23.58	22.73	23.14	23.53	21.48	23.74	22.35	23.14	22.27	22.90	25.29	23.60	27.09	23.65	28.26					
24: OsGA2ox8	26.95	24.12	26.30	25.33	27.78	24.76	25.40	24.67	24.58	24.60	24.92	23.64	25.63	23.49	25.32	24.51	23.51	26.73	25.33	27.39	26.44	29.68	84.78				
25: OsGA2ox6	27.12	25.57	25.32	26.49	28.31	25.08	25.08	24.18	24.09	23.81	23.81	22.29	24.21	23.57	22.88	23.47	24.14	26.73	24.25	25.08	24.25	26.50	64.56	68.12			
26: AtGA2ox8	26.35	23.75	20.47	24.32	25.70	22.80	24.75	24.67	24.24	25.57	25.57	25.66	23.70	25.66	23.13	25.75	21.68	23.70	27.15	30.03	29.41	28.78	50.55	54.49	50.00		
27: AtGA2ox7	23.59	23.68	23.43	23.57	26.51	24.92	23.61	25.17	25.75	24.92	25.24	25.57	25.64	25.89	23.62	25.00	23.62	24.35	25.68	26.51	27.49	26.95	37.00	39.64	39.04	42.73	
